# Match-day peaks and post-match compensation: divergent weekly load profiles of starters vs. non-starters in professional women's soccer

**DOI:** 10.3389/fspor.2026.1746747

**Published:** 2026-04-17

**Authors:** Pedro Schons, Artur Avelino Birk Preissler, Suellen dos Santos Ramos, Adam Kawczyński, Aleksandra Kisilewicz, Robert Trybulski, Filipe Manuel Clemente

**Affiliations:** 1 Faculdade SOGIPA, Porto Alegre, RS, Brazil; 2 School of Physical Education, Physiotherapy and Dance, Swimming Center, Federal University of Rio Grande do Sul, Porto Alegre, RS, Brazil; 3 Polytechnic Institute of Viana do Castelo, SPRINT, Viana do Castelo, Portugal; 4 Faculty of Medicine, Wrocław University of Science and Technology, Wrocław, Poland; 5 Faculty of Medicine, Katowice Business University, Katowice, Poland; 6 Provita Medical Centre, Żory, Poland; 7 Gdansk University of Physical Education and Sport, Gdańsk, Poland; 8 The Sport Physical Activity and Health Research & Innovation Center, Coimbra, Portugal; 9 Applied Research Institute, Polytechnic University of Coimbra, Coimbra, Portugal

**Keywords:** distance, female, football, GPS, training

## Abstract

**Introduction:**

Recent evidence indicates differences in training and match loads between starters and non-starters in women's soccer, but little is known about players in Brazil's elite leagues. This study aimed to compare the external and internal loads of professional women's soccer players classified as starters and non-starters during training sessions and matches.

**Methods:**

Twenty-nine players from a professional team competing in the Campeonato Brasileiro Série A1 (64.1 ± 6.8 kg; 167.3 ± 6.1 cm) were monitored. Internal load was assessed using the rating of perceived exertion (RPE), while external load variables included total distance, distance in different speed zones, high-speed running distance, number of sprints, maximum speed, accelerations and decelerations, recorded via Global Positioning System devices. Independent t-tests and Mann–Whitney *U* tests compared groups.

**Results:**

Starters accumulated higher match-day (MD) loads, with greater total distance (9999.0 ± 1205.7 vs. 5141.1 ± 1290.5 m; *p*<0.01) and RPE (8.0 [7.0 − 9.5] vs. 4.5 [4.0 − 5.5] A.U.; *p*<0.01). Conversely, non-starters presented higher values after matches, covering more distance on MD+1 (4655.1 ± 996.1 vs. 2390.4 ± 814.4 m; *p*<0.01) and MD+2 (4210.0 ± 992.0 vs. 2979.4 ± 526.7 m; *p*<0.01), along with higher RPE (7.0 [7.0–7.4] vs. 3.5 [2.5–5.0] A.U.; *p*<0.01, and 6.5 [6.0–7.0] vs. 4.0 [3.5–5.0] A.U.; *p*<0.01).

**Discussion:**

As conclusion, starters concentrated peak loads on MD, whereas non-starters accumulated higher loads on MD+1/MD+2. These findings support individualized post-match compensation for non-starters and recovery-focused management for starters.

## 1 Introduction

As women's soccer has professionalized, match play has been characterized by substantial locomotor and neuromuscular demands (e.g., high-speed running, sprinting, and frequent accelerations/decelerations), increasing the importance of systematically quantifying players' exposure to these demands (1). Elite women's soccer is increasingly characterized by dense competitive calendars and shorter recovery windows between matches, which can meaningfully affect recovery and performance capacity (2). In parallel, women's programs have expanded the use of structured athlete-monitoring approaches to quantify training and match demands (3). In practice, this commonly involves integrating external-load metrics derived from wearable microtechnology [e.g., Global Navigation Satellite System (GNSS)] with internal-load indices such as session rating of perceived exertion (RPE) (4). These global developments have increased the need to manage readiness across the full squad, particularly because starters and non-starters may accumulate substantially different load profiles across the microcycle (5). This need is amplified in squad-based sports because match participation is inherently uneven since starters typically accumulate greater match exposure, whereas non-starters may require compensatory stimuli to maintain fitness and readiness (6). Within this broader international landscape, Brazil has also accelerated investment and organizational development in the women's game, as the incentives provided by Confederación Sudamericana de Fútbol (CONMEBOL) have increased visibility (7), competitiveness, and the number of tournaments. This evolving competitive environment increases demands on coaching and performance staffs due to rising match and training loads. Despite the widespread adoption of monitoring, the evidence base remains limited and context-dependent regarding whether and on which microcycle days internal and external loads meaningfully diverge between starters and non-starters, particularly when both training and match demands are considered together (8).

External load in soccer refers to objective, movement-derived indicators of the work performed in training and matches, and, critically, these indicators are used not only to describe demands but also to plan and evaluate training prescription relative to match requirements (9). Accordingly, GNSS tracking is widely used to quantify the volume and intensity distribution of field-based work, enabling coaches to design sessions that reproduce (or deliberately undercut) match locomotor profiles depending on the day of the microcycle and the intended stimulus (9). From a practical prescription perspective, total distance primarily reflects overall running volume, while distance-per-minute (or comparable relative metrics) provides a simple index of locomotor intensity that practitioners can manipulate via drill duration, work–rest structure, and pitch size (9). Distance accumulated in higher-speed zones (high-speed running and sprinting), together with peak/maximal speed, is particularly relevant for ensuring adequate exposure to high-velocity actions that underpin match performance and that may be under-dosed in some training weeks without deliberate programming (10). Consistent with this, planned near-maximal sprint exposure before competition may reduce hamstring injury risk in elite soccer (11). In parallel, acceleration and deceleration counts capture a substantial component of “mechanical” (neuromuscular) load that is highly sensitive to drill constraints; for example, small-sided games and changes in player numbers/space can markedly increase or decrease acceleration–deceleration demands and are therefore actionable levers for prescription (12). Thus, the combined interpretation of distance-based (volume/intensity) and acceleration–deceleration (mechanical) metrics supports more precise, target-driven session design (e.g., conditioning-oriented days vs. tapering/recovery days) and can also guide individualized “top-up” or compensatory work when match exposure is limited (9). In addition, it has become standard practice to monitor internal load as the psycho-physiological response to imposed external demands, with session-RPE providing a validated, practical method that captures perceived intensity integrated over session duration (13). Although elite teams increasingly use these complementary tools, it remains unclear whether the resulting load-management decisions sufficiently balance exposure between starters and non-starters, a question that is directly relevant because both excessive and insufficient loading can plausibly compromise readiness, performance capacity, and well-being across a congested season.

Recent studies have reported significant differences in training and match loads between starters and non-starters in women's soccer (6, 8, 14). However, the direction and magnitude of these differences are inconsistent across studies and competitive contexts (6). This lack of convergence likely reflects methodological and contextual heterogeneity, including differences in (i) the observation window (e.g., pre-season vs. in-season, or microcycle-level vs. season-accumulated load), (ii) operational definitions of “starter”/“non-starter” (including minimum match-minutes criteria), (iii) the selection and processing of internal/external load metrics, and (iv) league- and team-specific factors (competition level, tactical model, travel, and scheduling) that shape weekly periodization (3, 15). In particular, external-load estimates vary with speed-zone definitions and sprint thresholds, limiting cross-study comparability (16). Additionally, much of the available evidence in women's soccer is based on single-team cohorts, limiting generalizability and increasing the likelihood that observed starter/non-starter differences are contingent on local training models and squad-management strategies (6). Consequently, important gaps remain to be addressed. To the best of our knowledge, no previous research has examined the differences between starters and non-starters among female soccer players competing in Brazil's elite leagues, which limits the applicability of current findings to this specific context. The practical relevance of the present study lies in providing data that may assist coaching staffs in designing compensatory training sessions (17) aimed at balancing training loads between starters and non-starters. These findings may inform performance and injury-prevention strategies in women's soccer.

Building on this mixed and context-dependent evidence, a methodological gap remains: much of the starter/non-starter literature in women's soccer has been generated in European elite contexts or North American collegiate settings, frequently using single-team cohorts (8). In addition, many studies compare groups using weekly or seasonal accumulated indicators (i.e., aggregated exposure across long observation windows), which can obscure when within the microcycle meaningful divergences emerge (6, 8). By contrast, day-relative microcycle approaches (match day (MD) minus/plus formats) are a well-established framework for quantifying how load is distributed across specific training days as competition approaches and recedes (18, 19). Within women's soccer, only a limited set of investigations have concurrently reported internal-load indices (e.g., RPE-based measures) and external-load measures across multiple pre- and post-match days, which constrains inference about the day-by-day coupling of perceived and locomotor/mechanical demands (20, 21). Furthermore, compensatory training for non-starters is widely discussed and empirically evaluated, yet it is not consistently integrated into starter/non-starter comparisons across the full microcycle (17). Finally, league-specific factors affect recovery and load management, underscoring the need for context-specific evidence (22). Against this backdrop (and to our knowledge, in the absence of equivalent analyses in Brazil's elite women's leagues) the present investigation provides structured, day-by-day, status-based comparisons (MD-3 to MD+3) within a Brazilian professional squad to inform how, and on which days, practitioners might calibrate recovery for starters and compensatory work for non-starters, thereby strengthening the ecological validity and practical transfer of load-management recommendations.

Given the relevance of this topic and its practical implications, the present study aimed to compare the external and internal loads of professional women's soccer players who were classified as starters and non-starters during training sessions and matches. The study hypothesized that starters would exhibit greater external and internal loads than non-starters during matches. However, it was expected that load differences during training sessions would vary according to the number of days relative to MD.

## 2 Material and methods

### 2.1 Study design and setting

This was a longitudinal study conducted within a single professional women's soccer club competing in Brazil's top division (Série A1). Data were collected during an in-season period in which the squad followed a standard seven-day microcycle anchored to the reference MD. All on-field team training sessions and official matches occurring from three days before to three days after each match (MD−3 to MD+3) were monitored. A total of 13 matches and their respective days before and after were analyzed. Data was included when players participated in training sessions and games with the team. The team exhibited a resting pattern of MD+1 or MD+2.Training content and scheduling were determined solely by the coaching staff according to the team's game model and competition calendar; the research team did not intervene in practice design. Environmental conditions, travel demands, and match location were not controlled during the study period.

Players were classified *post hoc* for each microcycle as starters or non-starters according to their starting status in the reference match. All outfield players available for full participation were eligible; goalkeepers were excluded. External load was captured during every monitored session and match using the club's validated 10-Hz GNSS tracking system worn in a fitted vest, and internal load was assessed via the session RPE collected using the club's standardized procedure after each session. Data were recorded as part of the team's established monitoring program and exported for analysis in anonymized form.

All participants were informed in advance about the risks and benefits of the assessments prior to data collection and signed an informed consent form. The study (No. 7.047.156) was approved by the Research Ethics Committee of the Pontifical Catholic University of Rio Grande do Sul (CEP-PUCRS) and adhered to the ethical principles of the Declaration of Helsinki of the World Medical Association.

### 2.2 Participants

A total of 29 professional women's soccer players participated in this study, presenting a mean age of 25.4 ± 5.5 years, a mean body mass of 64.1 ± 6.8 kg, and a mean height of 167.3 ± 6.1 cm. With respect to playing positions, 28% were central defenders (*n* = 8), 14% were full-backs (*n* = 4), 34% were midfielders (*n* = 10), and 24% were forwards (*n* = 7). The sample size was determined by convenience, based on the number of outfield players available on the participating team, considering only those eligible for official training sessions and matches during the season. The athletes belonged to a professional team competing in the Brazilian Women's Championship Série A1 and were assessed during the competitive period. Players followed regular training sessions according to the club's seasonal schedule. Eligibility criteria included: (i) regular participation in the team's training sessions and official competitions, and (ii) medical clearance from the club's medical department. Players who were injured or unable to train or compete during the data collection period were excluded, as well as goalkeepers, due to their position-specific movement profiles and bespoke training content.

### 2.3 Procedures

Initially, players were invited to participate in the study and received detailed explanations regarding its procedures and methodologies. After acknowledging full understanding of the information provided, they signed the informed consent form. Before data collection began, a familiarization session was conducted with all equipment and instruments used in the study, including the GNSS device and the RPE scale. Players were instructed to maintain their regular training and dietary routines throughout the study period. Information regarding age, body mass, height, and playing position was obtained at the beginning of the competitive phase. The GNSS device was fitted prior to the start of each training session or match to assess external load, while the RPE scale was completed after the end of each session. All data collection procedures were performed by the coaching staff and supervised by the research team. RPE was collected individually by the same member of the coaching staff after each session. Players were blinded to the final objective of the study. For each day, the median value of each variable was calculated for every player, and these values were used as reference points in the subsequent analyses.

### 2.4 Match status (starter or non-starter)

Players were monitored throughout the competitive period. Data regarding match status were obtained from the official match reports published on the Brazilian Football Confederation (CBF) website. Players were classified as starters if they began the match in the starting lineup and participated for at least 45 min. After collecting this information, an analysis was conducted to determine the number of matches in which each player was classified as a starter. The distribution of these values was used to calculate the median number of starting appearances within the group. This median value served as an objective cutoff point for classifying players as starters—those with a number of starts above the group median—and non-starters—those with a number of starts below the group median. This classification was applied consistently throughout the season for the analysis of both training sessions and official matches. This classification was adopted to provide stability across the competitive period; however, some players may have alternated between starter and non-starter roles across different microcycles.

### 2.5 Rate of perceived exertion (RPE)

Subjective perception of effort was assessed using the Borg CR-10 scale (0 = “rest”; 10 = “maximal”) to quantify internal load (23). Players recorded their responses in an electronic form after each training session and match. RPE was collected between 10 and 15 min after the end of each training session or match. This time window was standardized to minimize the influence of the final actions of training sessions and matches and to ensure external validity of the procedure. They were instructed to indicate the value that best represented their overall exertion during the activity, considering the session as a whole. RPE was collected individually by the same member of the coaching staff after each session under the supervision of the research team, and all data were stored in an electronic database for subsequent analysis.

### 2.6 Global navigation satellite system (GNSS)

External load data were collected using the Catapult GNSS system (Catapult Sports, Melbourne, Australia), operating at a sampling frequency of 10 Hz. The devices were positioned on the upper back of the players and secured using manufacturer-provided vests to ensure stability during movement. The 10 Hz Catapult GNSS has demonstrated good validity and interunit reliability in team-sport movement assessments, with a typical error of measurement of ≈1.3% for total distance and error increasing as speed rises (%TEM 0.8–19.9), while outperforming 15 Hz models for most metrics (24). For instantaneous velocity during acceleration and deceleration, 10 Hz units are two to three times more accurate than 5 Hz, showing validity coefficients of variation of 3.1%–11.3% and reliability coefficients of variation of 1.9%–6.0% (25).

The units were activated 10 min before the start of each session to allow signal stabilization and proper sensor calibration. The system automatically recorded the following metrics during all training sessions and official matches: total distance covered, distance covered within specific speed zones (≤7 km/h; 7–13 km/h; 13–19 km/h; 19–23 km/h; >23 km/h) (26), high-speed running distance (>19 km/h), number of sprints (>23 km/h), peak instantaneous speed (km/h), and number of accelerations (>2 m/s²) and decelerations (<2 m/s²). Speed zones were defined according to reference thresholds adopted by the Fédération Internationale de Football Association (26). After each session, data were downloaded using the Catapult OpenField software and stored in an electronic database for subsequent analysis.

### 2.7 Statistical analysis

Data were analyzed using descriptive statistics, including mean, standard deviation, and 95% confidence intervals. RPE data were treated as categorical variables; therefore, they are presented as median, interquartile range (25th–75th percentiles), location parameter, and 95% confidence intervals for the location parameter. Median values were used to classify players as starters or non-starters and to characterize the behavior of each variable for individual players relative to MD. Independent *t*-tests were employed to compare internal and external loads between starters and non-starters, with statistical significance set at α < 0.05. For variables that did not meet normality assumptions, the Mann–Whitney *U*-test was applied. Effect sizes were calculated using Cohen's d and qualitatively interpreted as: trivial (<0.19), small (0.20–0.49), medium (0.50–0.79), large (0.80–1.29), or very large (≥1.30) (27–29). All analyses were performed using SPSS software (v.22.0; IBM Corp., Armonk, NY, USA).

## 3 Results

The total number of observations recorded for each relative match day was: MD−3 (*n* = 217), MD−2 (*n* = 252), MD−1 (*n* = 227), MD (*n* = 235), MD+1 (*n* = 37), MD+2 (*n* = 132), and MD+3 (*n* = 193). On MD-3, starters covered a greater distance at ≤7 km/h compared with non-starters (*p* < 0.01; large effect size) (Table 1). Similarly, on MD-2, starters covered a greater distance at ≤7 km/h (*p* = 0.02; large effect size); however, non-starters covered a greater distance at 7–13 km/h (*p* = 0.04; medium effect size) (Table 2). On MD-1, no significant differences were observed between starters and non-starters for any internal or external load variables (all *p* > 0.05) (Table 3).

**Table 1:** Comparison of internal and external loads between starter and non-starter players in professional female soccer during training sessions conducted three days prior to a match (MD-3).

Variable	Group	*N*	Mean	SD	Mean difference	95% confidence interval		*p*	Statistical test	Effect size (Cohen's d)	95% confidence interval	
						Lower	Upper				Lower	Upper
RPE (A.U.)*	S	11	6.0	5.0–6.0	1.00	−0.50	1.00	0.23	^MWU^	0.53	−0.32	1.35
	NS	13	5.0	5.0–6.0								
Total distance covered (m)	S	11	4,544.0	379.4	238.20	−426.43	902.84	0.47	^ST^	0.28	−0.49	1.04
	NS	18	4,305.8	1,026.2								
Total high-speed running distance (m)	S	11	252.5	94.5	−26.04	−168.78	116.71	0.61	^MWU^	−0.14	−0.89	0.61
	NS	18	278.5	217.3								
Number of sprints (n)	S	11	8.4	2.9	−0.98	−5.93	3.97	0.50	^MWU^	−0.16	−0.90	0.60
	NS	18	9.4	7.6								
Number of accelerations (n)	S	11	103.8	13.9	20.19	−1.05	41.43	0.06	^ST^	0.75	−0.09	1.55
	NS	18	83.6	32.4								
Number of decelerations (n)	S	11	103.8	13.5	17.38	−4.20	38.97	0.06	^ST^	0.63	−0.18	1.42
	NS	18	86.4	33.1								
Maximum speed (km/h)	S	11	26.0	1.0	0.97	−0.81	2.75	0.52	^MWU^	0.43	−0.36	1.19
	NS	18	25.0	2.7								
Distance ≤ 7 km/h (m)	S	11	2,329.6	146.9	391.04	99.73	682.35	**<0** **.** **01**	^ST^	1.05	0.16	1.91
	NS	18	1,938.6	453.8								
Distance between 7 and 13 km/h (m)	S	11	1,270.4	138.5	−56.47	−485.56	372.63	0.36	^MWU^	−0.10	−0.85	0.65
	NS	18	1,326.9	680.4								
Distance between 13 and 19 km/h (m)	S	11	692.3	196.9	13.42	−253.02	279.87	0.92	^ST^	0.04	−0.71	0.79
	NS	18	678.8	400.1								
Distance between 19 and 23 km/h (m)	S	11	178.7	55.4	−37.55	−167.36	92.26	0.81	^MWU^	−0.23	−0.98	0.54
	NS	18	216.3	204.0								
Distance > 23 km/h (m)	S	11	68.3	48.4	22.44	−11.98	56.85	0.19	^MWU^	0.51	−0.28	1.28
	NS	18	45.8	40.9								

* Categorical variable: median, interquartile range (25th–75th percentiles), location parameter, and 95% confidence interval for the location parameter; RPE, rating of perceived exertion; A.U., arbitrary units; S, starter; NS, non-starter; N, number of players; SD, standard deviation; MWU, Mann–Whitney *U*-test; ST = Student's *t*-test.

Bold values indicate statistically significant differences (*p* < 0.05).

**Table 2:** Comparison of internal and external loads between starter and non-starter players in professional female soccer during training sessions conducted two days prior to a match (MD-2).

Variable	Group	*N*	Mean	SD	Mean difference	95% confidence interval		*p*	Statistical test	Effect Size (Cohen's d)	95% confidence interval	
						Lower	Upper				Lower	Upper
RPE (A.U.)*	S	11	4.0	4.0–4.3	0.00	−0.00	1.00	0.24	^MWU^	0.41	−0.42	1.22
	NS	13	4.0	3.0–4.0								
Total distance covered (m)	S	11	3,303.4	249.4	0.55	−197.96	199.06	1.00	^ST^	0.00	−0.77	0.77
	NS	16	3,302.8	243.9								
Total high-speed running distance (m)	S	11	66.6	33.1	17.81	−5.28	40.90	0.13	^ST^	0.62	−0.20	1.42
	NS	16	48.8	25.2								
Number of sprints (n)	S	11	1.5	1.0	0.36	−0.44	1.16	0.30	^MWU^	0.37	−0.43	1.14
	NS	16	1.1	1.0								
Number of accelerations (n)	S	11	89.6	9.8	−3.68	−12.69	5.34	0.41	^ST^	−0.33	−1.10	0.46
	NS	16	93.3	12.0								
Number of decelerations (n)	S	11	87.5	11.3	−6.13	−16.46	4.21	0.23	^ST^	−0.48	−1.26	0.33
	NS	16	93.6	13.7								
Maximum speed (km/h)	S	11	23.0	1.2	0.57	−0.32	1.47	0.20	^ST^	0.52	−0.30	1.30
	NS	16	22.4	1.1								
Distance ≤ 7 km/h (m)	S	11	2,103.2	155.4	220.26	16.14	424.37	**0** **.** **02**	^ST^	0.87	0.00	1.71
	NS	16	1,882.9	301.0								
Distance between 7 and 13 km/h (m)	S	11	843.6	79.4	−108.90	−245.32	27.51	**0** **.** **04**	^MWU^	−0.64	−1.45	0.19
	NS	16	952.5	208.5								
Distance between 13 and 19 km/h (m)	S	11	406.6	69.3	−32.58	−118.28	53.13	0.44	^ST^	−0.31	−1.08	0.48
	NS	16	439.2	124.9								
Distance between 19 and 23 km/h (m)	S	11	59.8	29.1	16.69	−3.22	36.59	0.10	^ST^	0.68	−0.16	1.48
	NS	16	43.1	21.2								
Distance > 23 km/h (m)	S	11	5.2	5.4	2.20	−1.21	5.60	0.45	^MWU^	0.52	−0.29	1.31
	NS	16	3.0	3.2								

* Categorical variable: median, interquartile range (25th–75th percentiles), location parameter, and 95% confidence interval for the location parameter; RPE, rating of perceived exertion; A.U., arbitrary units; S, starter; NS, non-starter; N, number of players; SD, standard deviation; MWU, Mann–Whitney *U*-test; ST, Student's *t*-test.

Bold values indicate statistically significant differences (*p* < 0.05).

**Table 3:** Comparison of internal and external loads between starter and non-starter players in professional female soccer during training sessions conducted one day prior to a match (MD-1).

Variable	Group	*N*	Mean	SD	Mean difference	95% confidence interval		*p*	Statistical test	Effect size (Cohen's d)	95% confidence interval	
						Lower	Upper				Lower	Upper
RPE (A.U.)*	S	11	3.0	3.0–4.0	0.00	−0.00	1.00	0.39	^MWU^	0.22	−0.59	1.03
	NS	13	3.0	3.0–3.0								
Total distance covered (m)	S	11	2,515.7	289.2	123.49	−162.68	409.65	0.38	^ST^	0.36	−0.45	1.16
	NS	14	2,392.2	379.8								
Total high-speed running distance (m)	S	11	54.6	46.6	−9.34	−56.80	38.11	0.85	^MWU^	−0.16	−0.95	0.63
	NS	14	63.9	63.8								
Number of sprints (n)	S	11	1.4	1.8	−0.74	−2.65	1.16	0.50	^MWU^	−0.33	−1.12	0.49
	NS	14	2.1	2.6								
Number of accelerations (n)	S	11	65.8	9.6	1.59	−10.05	13.24	0.78	^ST^	0.11	−0.68	0.90
	NS	14	64.2	16.6								
Number of decelerations (n)	S	11	61.3	8.7	−3.05	−15.44	9.34	0.62	^ST^	−0.21	−0.99	0.60
	NS	14	64.3	18.2								
Maximum speed (km/h)	S	11	22.5	1.8	0.11	−1.48	1.69	0.89	^ST^	0.06	−0.73	0.85
	NS	14	22.4	2.0								
Distance ≤ 7 km/h (m)	S	11	1,686.9	123.4	59.88	−57.04	176.79	0.30	^ST^	0.43	−0.39	1.23
	NS	14	1,627.1	152.0								
Distance between 7 and 13 km/h (m)	S	11	509.1	83.4	4.11	−82.75	90.97	0.92	^ST^	0.04	−0.75	0.83
	NS	14	504.9	117.7								
Distance between 13 and 19 km/h (m)	S	11	227.1	75.4	−1.65	−80.19	76.88	0.97	^ST^	−0.02	−0.81	0.77
	NS	14	228.7	106.5								
Distance between 19 and 23 km/h (m)	S	11	48.0	37.8	−5.13	−41.58	31.32	0.85	^MWU^	−0.12	−0.91	0.68
	NS	14	53.1	47.8								
Distance > 23 km/h (m)	S	11	6.2	9.3	0.43	−6.26	7.12	0.98	^MWU^	0.05	−0.74	0.84
	NS	14	5.8	6.9								

* Categorical variable: median, interquartile range (25th–75th percentiles), location parameter, and 95% confidence interval for the location parameter; RPE, rating of perceived exertion; A.U., arbitrary units; S, starter; NS, non-starter; N, number of players; SD, standard deviation; MWU, Mann–Whitney *U*-test; ST, Student's *t*-test.

Bold values indicate statistically significant differences (p < 0.05).

On MD, starters exhibited significantly higher internal and external load values than non-starters across all variables (*p* < 0.05; large to very large effect sizes) (Table 4).

**Table 4:** Comparison of internal and external loads between starter and non-starter players in professional female soccer during the match day (MD).

Variable	Group	*N*	Mean	SD	Mean difference	95% confidence interval		*p*	Statistical test	Effect size (Cohen's d)	95% confidence interval	
						Lower	Upper				Lower	Upper
RPE (A.U.)*	S	11	8.0	7.0–9.5	3.00	2.00	4.50	**<0** **.** **01**	^MWU^	2.25	0.97	3.49
	NS	13	4.5	4.0–5.5								
Total distance covered (m)	S	11	9,999.0	1,205.7	4,857.83	3,793.55	5,922.10	**<0** **.** **01**	^ST^	3.88	2.01	5.72
	NS	13	5,141.1	1,290.5								
Total high-speed running distance (m)	S	11	515.0	141.4	235.41	120.71	350.10	**<0** **.** **01**	^ST^	1.74	0.62	2.82
	NS	13	279.6	129.4								
Number of sprints (n)	S	11	16.9	5.1	8.10	4.06	12.14	**<0** **.** **01**	^ST^	1.70	0.59	2.77
	NS	13	8.8	4.5								
Number of accelerations (n)	S	11	196.4	32.2	80.64	55.88	105.40	**<0** **.** **01**	^ST^	2.77	1.31	4.19
	NS	13	115.8	26.4								
Number of decelerations (n)	S	11	201.9	26.5	79.98	57.50	102.45	**<0** **.** **01**	^ST^	3.02	1.47	4.54
	NS	13	121.9	26.4								
Maximum speed (km/h)	S	11	26.9	1.1	1.02	0.06	1.98	**0** **.** **04**	^ST^	0.90	−0.01	1.78
	NS	13	25.9	1.2								
Distance ≤ 7 km/h (m)	S	11	4,580.3	646.3	1,615.64	1,155.06	2,076.23	**<0** **.** **01**	^ST^	2.98	1.44	4.48
	NS	13	2,964.7	436.7								
Distance between 7 and 13 km/h (m)	S	11	3,188.7	438.2	1,960.91	1,543.86	2,377.97	**<0** **.** **01**	^ST^	4.00	2.08	5.88
	NS	13	1,227.8	530.8								
Distance between 13 and 19 km/h (m)	S	11	1,618.1	272.4	927.49	677.44	1,177.55	**<0** **.** **01**	^ST^	3.15	1.55	4.71
	NS	13	690.6	311.4								
Distance between 19 and 23 km/h (m)	S	11	370.0	97.7	175.64	96.26	255.02	**<0** **.** **01**	^ST^	1.88	0.72	3.00
	NS	13	194.3	89.8								
Distance > 23 km/h (m)	S	11	131.6	68.1	57.83	8.49	107.17	**0** **.** **02**	^MWU^	1.00	0.07	1.89
	NS	13	73.8	48.1								

* Categorical variable: median, interquartile range (25th–75th percentiles), location parameter, and 95% confidence interval for the location parameter; RPE, rating of perceived exertion; A.U., arbitrary units; S, starter; NS, non-starter; N, number of players; SD, standard deviation; MWU, Mann–Whitney *U*-test; ST, Student's *t*-test.

Bold values indicate statistically significant differences (*p* < 0.05).

On MD+1, non-starters demonstrated higher internal and external load values than starters (*p* < 0.05; large to very large effect sizes), except for the distance covered at speeds > 23 km/h (*p* = 0.09) (Table 5). Similarly, on MD+2, non-starters again exhibited higher internal and external load values than starters (*p* < 0.05; medium to very large effect sizes), except for the number of sprints (*p* = 0.08) (Table 6). On MD+3, no significant differences were observed between starters and non-starters for any internal or external load variables (Table 7).

**Table 5:** Comparison of internal and external loads between starter and non-starter players in professional female soccer during training sessions conducted one day after a match (MD+1).

Variable	Group	*N*	Mean	SD	Mean difference	95% confidence interval		*p*	Statistical test	Effect size (Cohen's d)	95% confidence interval	
						Lower	Upper				Lower	Upper
RPE (A.U.)*	S	11	3.5	2.5–5.0	−3.50	−5.00	−1.50	**<0** **.** **01**	^MWU^	−1.69	−2.79	−0.54
	NS	10	7.0	7.0–7.4								
Total distance covered (m)	S	11	2,390.4	814.4	−2,264.62	−3,092.29	−1,436.95	**<0** **.** **01**	^MWU^	−2.50	−3.86	−1.10
	NS	10	4,655.1	996.1								
Total high-speed running distance (m)	S	11	19.7	37.0	−41.91	−76.38	−7.44	**0** **.** **01**	^MWU^	−1.11	−2.07	−0.11
	NS	10	61.6	38.5								
Number of sprints (n)	S	11	0.6	1.2	−1.06	−2.29	0.17	**0** **.** **04**	^MWU^	−0.79	−1.69	0.15
	NS	10	1.7	1.5								
Number of accelerations (n)	S	11	30.4	43.3	−130.34	−179.05	−81.63	**<0** **.** **01**	^MWU^	−2.45	−3.78	−1.06
	NS	10	160.8	62.5								
Number of decelerations (n)	S	11	33.6	46.1	−135.26	−187.05	−83.47	**<0** **.** **01**	^MWU^	−2.39	−3.71	−1.02
	NS	10	168.9	66.4								
Maximum speed (km/h)	S	11	12.5	3.4	−8.41	−11.67	−5.16	**<0** **.** **01**	^MWU^	−2.36	−3.67	−1.01
	NS	10	20.9	3.7								
Distance ≤ 7 km/h (m)	S	11	1,083.6	410.9	−1,144.11	−1,617.20	−671.03	**<0** **.** **01**	^MWU^	−2.21	−3.47	−0.91
	NS	10	2,227.7	614.3								
Distance between 7 and 13 km/h (m)	S	11	1,187.4	445.5	−632.54	−960.34	−304.74	**<0** **.** **01**	^ST^	−1.76	−2.89	−0.59
	NS	10	1,819.9	225.3								
Distance between 13 and 19 km/h (m)	S	11	99.8	178.5	−446.07	−665.39	−226.75	**<0** **.** **01**	^MWU^	−1.86	−3.01	−0.66
	NS	10	545.8	293.3								
Distance between 19 and 23 km/h (m)	S	11	16.7	30.1	−42.09	−72.79	−11.38	**0** **.** **01**	^MWU^	−1.25	−2.25	−0.22
	NS	10	58.8	37.1								
Distance > 23 km/h (m)	S	11	3.0	7.9	0.17	−5.50	5.84	0.09	^MWU^	0.03	−0.83	0.88
	NS	10	2.9	3.3								

* Categorical variable: median, interquartile range (25th–75th percentiles), location parameter, and 95% confidence interval for the location parameter; RPE, rating of perceived exertion; A.U., arbitrary units; S, starter; NS, non-starter; N, number of players; SD, standard deviation; MWU, Mann–Whitney *U*-test; ST, Student's *t*-test.

Bold values indicate statistically significant differences (*p* < 0.05).

**Table 6:** Comparison of internal and external loads between starter and non-starter players in professional female soccer during training sessions conducted two days after a match (MD+2).

Variable	Group	*N*	Mean	SD	Mean difference	95% confidence interval		*p*	Statistical test	Effect size (Cohen's d)	95% confidence interval	
						Lower	Upper				Lower	Upper
RPE (A.U.)*	S	11	4.0	3.5–5.0	−2.50	−3.50	−1.00	**<0** **.** **01**	^MWU^	−1.65	−2.70	−0.55
	NS	13	6.5	6.0–7.0								
Total distance covered (m)	S	11	2,979.4	526.7	−1,230.60	−1,906.18	−555.02	**<0** **.** **01**	^MWU^	−1.47	−2.44	−0.45
	NS	16	4,210.0	992.0								
Total high-speed running distance (m)	S	11	26.4	26.7	−55.43	−101.67	−9.19	**0** **.** **01**	^MWU^	−0.97	−1.82	−0.07
	NS	16	81.8	70.7								
Number of sprints (n)	S	11	0.6	0.9	−1.67	−3.60	0.26	0.08	^MWU^	−0.70	−1.51	0.14
	NS	16	2.2	3.0								
Number of accelerations (n)	S	11	79.4	46.6	−43.84	−78.24	−9.44	**0** **.** **02**	^ST^	−1.03	−1.90	−0.12
	NS	16	123.3	39.8								
Number of decelerations (n)	S	11	73.2	51.1	−58.29	−93.06	−23.52	**<0** **.** **01**	^ST^	−1.35	−2.30	−0.37
	NS	16	131.5	36.8								
Maximum speed (km/h)	S	11	20.1	2.1	−2.51	−4.08	−0.94	**<0** **.** **01**	^ST^	−1.29	−2.22	−0.32
	NS	16	22.6	1.8								
Distance ≤ 7 km/h (m)	S	11	1,487.0	286.7	−573.85	−962.07	−185.64	**<0** **.** **01**	^ST^	−1.19	−2.10	−0.25
	NS	16	2,060.8	575.5								
Distance between 7 and 13 km/h (m)	S	11	1,081.4	278.5	−405.28	−710.22	−100.34	**<0** **.** **01**	^MWU^	−1.07	−1.95	−0.16
	NS	16	1,486.7	431.8								
Distance between 13 and 19 km/h (m)	S	11	274.3	137.8	−320.70	−480.76	−160.63	**<0** **.** **01**	^ST^	−1.62	−2.63	−0.56
	NS	16	595.0	230.1								
Distance between 19 and 23 km/h (m)	S	11	25.5	26.4	−39.26	−70.58	−7.94	**0** **.** **02**	^ST^	−1.01	−1.88	−0.11
	NS	16	64.8	45.3								
Distance > 23 km/h (m)	S	11	0.6	1.3	−11.44	−29.43	6.55	**0** **.** **03**	^MWU^	−0.51	−1.30	0.30
	NS	16	12.0	28.8								

* Categorical variable: median, interquartile range (25th–75th percentiles), location parameter, and 95% confidence interval for the location parameter; RPE, rating of perceived exertion; A.U., arbitrary units; S, starter; NS, non-starter; N, number of players; SD, standard deviation; MWU, Mann–Whitney *U*-test; ST = Student's *t*-test.

Bold values indicate statistically significant differences (*p* < 0.05).

**Table 7:** Comparison of internal and external loads between starter and non-starter players in professional female soccer during training sessions conducted three days after a match (MD+3).

Variable	Group	*N*	Mean	SD	Mean difference	95% confidence interval		*p*	Statistical test	Effect size (Cohen's d)	95% confidence interval	
						Lower	Upper				Lower	Upper
RPE (A.U.)*	S	11	5.5	5.3–6.3	−0.00	−1.00	0.50	0.47	^MWU^	−0.32	−1.13	0.50
	NS	13	6.0	5.0–7.0								
Total distance covered (m)	S	11	4,835.2	436.3	53.66	−327.51	434.84	0.78	^ST^	0.11	−0.65	0.87
	NS	17	4,781.5	504.2								
Total high-speed running distance (m)	S	11	185.2	84.4	−16.93	−93.41	59.55	0.68	^MWU^	−0.18	−0.93	0.59
	NS	17	202.2	102.8								
Number of sprints (n)	S	11	6.1	2.9	−0.72	−3.17	1.73	0.55	^ST^	−0.23	−0.99	0.54
	NS	17	6.8	3.2								
Number of accelerations (n)	S	11	119.1	16.2	3.40	−14.92	21.73	0.78	^MWU^	0.15	−0.62	0.91
	NS	17	115.7	26.5								
Number of decelerations (n)	S	11	121.6	19.4	−3.16	−22.11	15.79	0.18	^MWU^	−0.13	−0.89	0.63
	NS	17	124.8	26.2								
Maximum speed (km/h)	S	11	26.0	1.5	0.10	−1.13	1.33	0.87	^ST^	0.07	−0.69	0.82
	NS	17	25.9	1.6								
Distance ≤ 7 km/h (m)	S	11	2,531.8	127.1	86.03	−90.83	262.89	0.33	^ST^	0.39	−0.40	1.15
	NS	17	2,445.8	265.1								
Distance between 7 and 13 km/h (m)	S	11	1,430.6	187.8	−33.84	−251.40	183.73	1.00	^MWU^	−0.12	−0.88	0.64
	NS	17	1,464.4	315.5								
Distance between 13 and 19 km/h (m)	S	11	663.9	140.7	27.79	−85.11	140.68	0.62	^ST^	0.20	−0.57	0.95
	NS	17	636.1	142.7								
Distance between 19 and 23 km/h (m)	S	11	130.5	46.5	−14.20	−62.22	33.81	0.55	^ST^	−0.24	−0.99	0.54
	NS	17	144.7	67.6								
Distance > 23 km/h (m)	S	11	49.0	40.1	−5.53	−40.22	29.17	0.64	^MWU^	−0.13	−0.88	0.64
	NS	17	54.5	45.7								

* Categorical variable: median, interquartile range (25th–75th percentiles), location parameter, and 95% confidence interval for the location parameter; RPE, rating of perceived exertion; A.U., arbitrary units; S, starter; NS, non-starter; N, number of players; SD, standard deviation; MWU, Mann–Whitney *U*-test; ST, Student's *t*-test.

Bold values indicate statistically significant differences (*p* < 0.05).

**Figure 1:**
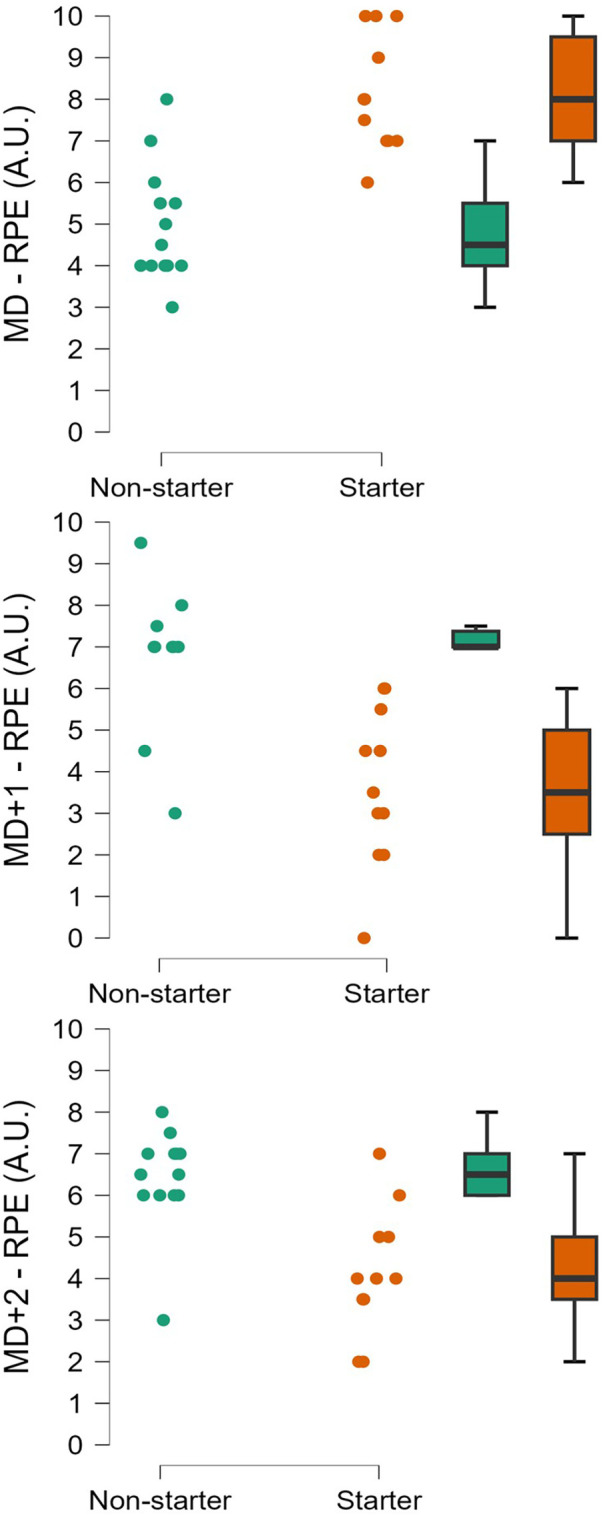
Rating of perceived exertion (RPE) for match day (MD), one day after (MD+1), and two days after (MD+2). Individual data points are presented alongside boxplots displaying the median and interquartile range (25th–75th percentiles). All comparisons showed significant differences (*p* < 0.05).

**Figure 2:**
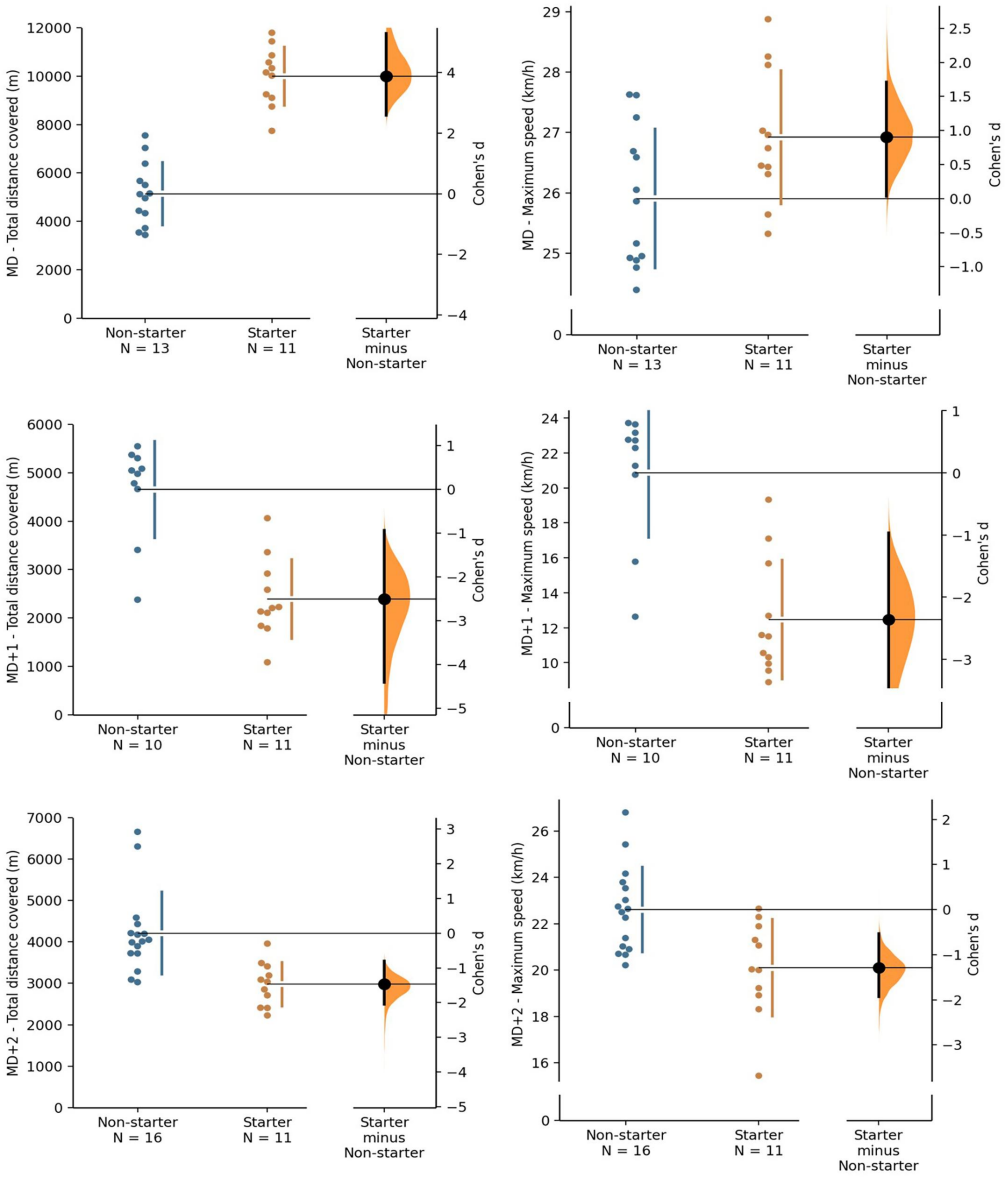
Total distance covered and maximum speed for match day (MD), one day after (MD+1), and two days after (MD+2). The Cohen's d between non-starter and starter is shown in the above Gardner-Altman estimation plot. Both groups are plotted on the left axes; the mean difference is plotted on a floating axes on the right as a bootstrap sampling distribution. The mean difference is depicted as a dot; the 95% confidence interval is indicated by the ends of the vertical error bar (28). All comparisons showed significant differences (*p* < 0.05).

Figure 1 presents the comparison of RPE between starters and non-starters on MD, MD+1, and MD+2. Figure 2 presents the comparison of total distance covered and maximum speed between starters and non-starters on MD, MD+1, and MD+2.

## 4 Discussion

The present study compared internal and external loads between starters and non-starters across the microcycle in a Brazilian professional women's soccer team. The principal findings were day-specific: starters accumulated substantially greater match-day (MD) loads across all variables, whereas non-starters accumulated higher external loads and reported higher RPE primarily on MD+1 and MD+2. Smaller and context-dependent differences were observed on MD-3 and MD-2, with broadly similar loads on MD-1 and MD+3. Our results indicate a practical microcycle distribution in which match exposure drives the weekly peak for starters and post-match compensation is used to partially offset reduced match minutes in non-starters.

A first main finding was that starters far exceeded non-starters on MD in total distance, distance in every speed zone, sprint metrics, accelerations/decelerations, and RPE. These outcomes align with prior reports that starters accrue greater match exposures and internal load than non-starters across a season in women's soccer. This pattern has been shown in Spanish first-division players for weekly internal workload (starters > non-starters) (6). Similar between-status disparities in accumulated distance and training load have also been described in collegiate women's soccer (starters > reserves) (8). Theoretically, the higher MD demands for starters reflect match selection itself and the well-documented locomotor and neuromuscular requirements of women's match play—substantial high-speed running, sprinting, and frequent acceleration/deceleration events—which are concentrated among those playing longer minutes (30, 31).

A second main finding was the absence of between-group differences on MD-1, with both starters and non-starters showing similar internal and external load profiles. This pattern is broadly consistent with in-season practices that taper or standardize the day-before session to emphasize tactical and set-piece preparation, as reported in elite women's soccer where MD-1 is typically shorter and less intense irrespective of starting status (6). Homogenized MD-1 content reflects the need to minimize residual fatigue before competition and prioritize freshness and tactical cohesion, which reduces scope for differentiating loads by starting status (6). The minimal differentiation on MD-1 implies that “exposure balancing” is unlikely to be efficiently achieved immediately pre-match and is better targeted earlier (MD-3/MD-2) and/or after the match (MD+1/MD+2), depending on the team's recovery model and match congestion.

A third main finding concerned small but significant differences earlier in the week: on MD-3 and MD-2, starters accumulated more low-speed distance (≤7 km/h), whereas non-starters covered more distance at 7–13 km/h on MD-2. These between-status differences were small and confined to specific speed zones, suggesting that earlier-week training content was broadly similar between groups in this squad, with only modest divergence in movement-intensity distribution. Such contrasts may reflect differences in drill role allocation, positional rehearsal time, or drill constraints (space, numbers, work–rest structure) that slightly shift continuous movement opportunities between groups. Comparable, microcycle-dependent fluctuations in women's elite squads have been observed during training camps and across in-season weeks (20, 32, 33). Subtle shifts in intensity bands can arise from training design emphasizing positional structures and game model tasks (e.g., larger spaces for starters' rehearsals vs. more continuous conditioning tasks for non-starters) and from the natural variability of acceleration-deceleration exposure, which is sensitive to drill constraints and change-of-direction density (34).

A fourth main finding was that non-starters attained greater loads and higher RPE on MD+1 and MD+2 across multiple variables, evidencing a compensatory strategy to offset lower match exposure. This pattern is practically important because compensatory strategies for female non-starters (including game-based formats) can increase post-match external load and can be selected specifically to better approximate match demands (17). This reflects emerging evidence that compensatory sessions for non-starters can substantially increase post-match external load and, when game-based formats are used, more closely approximate match demands in women's teams (17). Furthermore, narrative and empirical work proposes structured compensatory training as essential to balance weekly loads and maintain fitness in players with fewer minutes (15). Increasing MD+1/MD+2 load in non-starters eventually mitigates the fitness and readiness gap created by reduced high-intensity match play while fitting the recovery needs of starters, who require lower post-match loads due to greater preceding neuromuscular strain and glycogen depletion (35). Thus, the combination of very high MD loads in starters and elevated MD+1/MD+2 loads in non-starters supports a “split-purpose” post-match period (recovery-emphasis for starters and targeted exposure for non-starters) rather than attempting to differentiate loads on MD-1.

The present dataset does not allow causal inference about coaching rationale, nor does it allow determination of whether compensatory sessions replicate match-like high-speed or neuromuscular demands; however, several plausible, evidence-consistent factors may explain why compensation is scheduled on MD+1/MD+2 rather than immediately post-match. One possibility is that match-play can induce neuromuscular performance decrements and muscle-damage markers that persist for at least 24–72 h in elite female players (36). Moreover, post-match best practice in soccer commonly prioritizes low-effort recovery domains (sleep, nutrition/rehydration, and other recovery modalities) in the immediate hours after competition, which may reduce feasibility or desirability of adding substantial additional field load the same day (37). Also, compensatory training for non-starters is frequently implemented on MD+1 in women's soccer interventions, which is consistent with the timing observed in this squad (17). Accordingly, these findings should be interpreted as descriptive of load distribution across the microcycle, our interpretation is that scheduling compensation on MD+1/MD+2 can be a defensible approach when it preserves immediate post-match recovery priorities and logistical constraints (late kick-off times, media duties, travel), while still allowing staff to deliver a targeted stimulus within the early post-match window (37). Notably, the median-based classification used in this study may not capture week-to-week role alternations; however, the consistent post-match microcycle structure likely accommodates this variability, and results reflect typical load-distribution patterns rather than match-by-match status.

A final main finding was the convergence of loads on MD+3, with no significant differences between groups. Similar equilibration later in the week has been described when teams periodize a return to more uniform training content as both starters and non-starters prepare for the subsequent match microcycle, thereby narrowing status-based disparities (6). This likely reflects recovery progression in starters and completion of compensatory stimuli in non-starters, leading to comparable readiness and load tolerance by the third day post-match.

This study was conducted within a single professional squad competing in Brazil's Série A1, which may limit generalizability to other competitive levels, leagues, or tactical models. Playing-position effects were not stratified, which may affect the precision and generalizability of load comparisons between starters and non-starters, given the position-dependent nature of speed-zone and acceleration–deceleration profiles in women's soccer (30). The limitation associated with independent group-level analyses due to the repeated-measures structure of the data was partially mitigated by the use of median values; however, this should be considered when interpreting the findings. Although 10 Hz GNSS is widely accepted, measurement error increases during very high accelerations, which should be acknowledged when interpreting acceleration measures (38). To strengthen practical transfer, future studies should replicate these day-relative comparisons across multiple Brazilian teams and competitive phases, stratify by position and contextual factors (opponent strength, match outcome, travel), and experimentally test compensatory designs (e.g., game-based vs. running-based) to determine which most effectively reproduces match-like high-speed and acceleration–deceleration demands for non-starters without compromising subsequent readiness.

## 5 Conclusion

The present study described day-relative internal and external load distribution across an in-season microcycle in a Brazilian professional women's soccer team. Starters concentrated peak demands on MD, whereas non-starters accumulated relatively higher loads on MD+1 and MD+2, with minimal between-status differences on MD-1 and convergence by MD+3. Practically, these findings support a polarized post-match approach, in specific, prioritize recovery for starters while prescribing structured compensatory sessions for non-starters to reduce exposure gaps without inflating pre-match load. These results should be interpreted in context, as data derive from a single squad and league, without stratification by position or situational factors. Future multi-team studies incorporating position- and context-specific modifiers are warranted to refine compensatory-training prescriptions across different competitive calendars.

## 6 Practical application

In this population, starters concentrated the highest internal and external loads on MD, whereas non-starters accumulated relatively higher loads on MD+1 and MD+2, with minimal status-based differences on MD-1 and convergence by MD+3. Accordingly, the findings support a polarized post-match microcycle logic in which recovery is emphasized for starters after competition while non-starters receive additional exposure on MD+1/MD+2 to reduce match-exposure gaps. This interpretation is consistent with experimental work in women's soccer showing that compensatory strategies (including game-based formats) can meaningfully increase non-starters' post-match external load and better approximate match demands (17).

When the objective is to preferentially increase neuromuscular (acceleration–deceleration) stimulus for non-starters, drill constraints can be adjusted (e.g., relative space, player numbers, and bout density/work–rest structure) to modulate acceleration-event exposure while maintaining tactical specificity (34). If practitioners use GNSS-derived acceleration and deceleration measures to monitor these “top-up” doses, measurement limitations at higher accelerations should be considered and device validity/reliability evidence should guide interpretation (38). Finally, given the observed minimal differentiation on MD-1 and MD+3 in this squad, more uniform loading on those days appears justifiable when the session aims are tactical cohesion, freshness, and readiness for the next fixture.

## Data Availability

The datasets presented in this article are not readily available because The data are part of the repository of a high-performance club and were made available exclusively for the purposes of this study. Requests to access the datasets should be directed to pedroschons@hotmail.com.
